# In Silico Prediction of the Metabolic Resistance of Vitamin D Analogs against CYP3A4 Metabolizing Enzyme

**DOI:** 10.3390/ijms23147845

**Published:** 2022-07-16

**Authors:** Teresa Żołek, Kaori Yasuda, Geoffrey Brown, Toshiyuki Sakaki, Andrzej Kutner

**Affiliations:** 1 Department of Organic Chemistry, Faculty of Pharmacy, Medical University of Warsaw, 1 Banacha, 02-097 Warsaw, Poland; 2 Department of Pharmaceutical Engineering, Toyama Prefectural University, Toyama 939-0398, Japan; kyasuda@pu-tayama.ac.jp (K.Y.); tsakaki@pu-toyama.ac.jp (T.S.); 3 School of Biomedical Sciences, Institute of Clinical Sciences, College of Medical and Dental Sciences, University of Birmingham, Edgbaston, Birmingham B15 2TT, UK; g.brown@bham.ac.uk; 4 Department of Bioanalysis and Drug Analysis, Faculty of Pharmacy, Medical University of Warsaw, 1 Banacha, 02-097 Warsaw, Poland; andrzej.kutner@wum.edu.pl

**Keywords:** vitamin D analogs, cytochrome P450 3A4, cytochrome P450 24A1, in silico prediction, molecular dynamics

## Abstract

The microsomal cytochrome P450 3A4 (CYP3A4) and mitochondrial cytochrome P450 24A1 (CYP24A1) hydroxylating enzymes both metabolize vitamin D and its analogs. The three-dimensional (3D) structure of the full-length native human CYP3A4 has been solved, but the respective structure of the main vitamin D hydroxylating CYP24A1 enzyme is unknown. The structures of recombinant CYP24A1 enzymes have been solved; however, from studies of the vitamin D receptor, the use of a truncated protein for docking studies of ligands led to incorrect results. As the structure of the native CYP3A4 protein is known, we performed rigid docking supported by molecular dynamic simulation using CYP3A4 to predict the metabolic conversion of analogs of 1,25-dihydroxyvitamin D2 (1,25D2). This is highly important to the design of novel vitamin D-based drug candidates of reasonable metabolic stability as CYP3A4 metabolizes ca. 50% of the drug substances. The use of the 3D structure data of human CYP3A4 has allowed us to explain the substantial differences in the metabolic conversion of the side-chain geometric analogs of 1,25D2. The calculated free enthalpy of the binding of an analog of 1,25D2 to CYP3A4 agreed with the experimentally observed conversion of the analog by CYP24A1. The metabolic conversion of an analog of 1,25D2 to the main vitamin D hydroxylating enzyme CYP24A1, of unknown 3D structure, can be explained by the binding strength of the analog to the known 3D structure of the CYP3A4 enzyme.

## 1. Introduction

**Figure 1:**
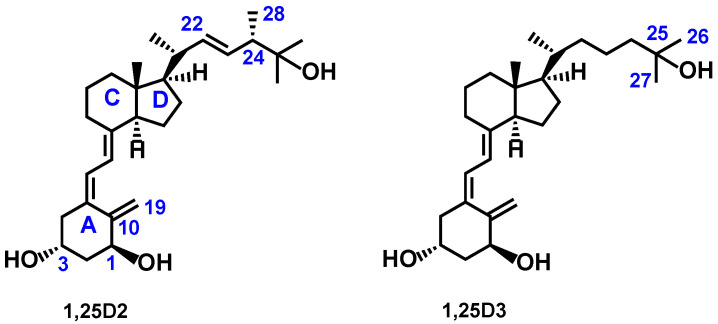
Structural formulas of 1α,25-dihydroxyergocalciferol (1,25-dihydroxyvitamin D_2_, 1,25D2) and 1α,25-dihydroxycholecalciferol (1,25-dihydroxyvitamin D_3_, 1,25D3).

1,25-dihydroxycholecalciferol (1,25-dihydroxyvitamin D_3_, calcitriol, 1,25D3) and 1,25-dihydroxyergocalciferol (1,25-dihydroxyvitamin D_2_, 1,25D2) (Figure 1) are no longer considered just as “vital amines” [1,2] that maintain the calcium and phosphate homeostasis [3], they are also pleiotropic hormones [4] that regulate key physiological processes [5,6]. Several active vitamin D metabolites, their precursors, and synthetic analogs are used as drug substances in the treatment of bone diseases (type I rickets, osteomalacia, hypoparathyroidism, pseudohypoparathyroidism, renal osteodystrophy, and osteoporosis), and hyperproliferative skin diseases such as psoriasis [7,8]. Efforts are now focused on fortifying standard anticancer chemotherapy by the addition of synthetic vitamin D analogs to improve efficacy and delay cancer recurrence [9]. The active forms of vitamin D_3_ (1,25D3) and D_2_ (1,25D2), jointly denoted as 1,25D, cannot be used for this purpose because they are highly calcaemic. They also have a low resistance to vitamin D metabolizing enzymes [10]. The current search is for vitamin D analogs of therapeutic potential against leukemia [7,11,12] and solid tumors, including breast [13], lung [14], prostate [15], colorectal [16,17], skin [18], and ovarian cancer [19]. We have developed analogs of 1,25D that exceed the potency of the parent 1,25D against cancer cells in vitro and in vivo, and that are less calcaemic and more enzymatically stable [10].

The rational design of vitamin D analogs is hampered by the lack of the 3D structure of the native full-length human vitamin D receptor (*h*VDR); the use of an engineered and truncated *h*VDRΔ [20] might result in a misleading outcome. For example, an early and controversial finding is that the *h*VDRΔ structure has an identical conformation, but that there is overlapping of all of the hydroxyls for 1,25D3 and two other analogs of very different structures when 1,25D3 and the analogs are bound to the ligand-binding pocket [21]. This intriguing finding has never been explained. Therefore, the use of the native integral vitamin D protein is recommended for the reliable design of analogs [22]. There is also the need to predict the metabolic resistance of potential anticancer analogs of 1,25D; this is important to a drug candidate prior to initiating a multistep synthesis followed by broad biological screening.

Cytochrome P450 hydroxylates vitamin D and its analogs. It also metabolizes several xenobiotics, including various drug substances. For instance, we postulated that our vitamin D analogs and the drug substance imatinib compete for the active site of the same cytochrome P450 enzyme [23]. This competition delays the deactivating metabolism of a vitamin D analog and prolongs the synergistic anticancer activity of both agents. Our concept has been confirmed recently [24] by studies that have shown that there is a pharmacodynamic synergy between ginsengoside Rh2 and 1,25D3 regarding the growth inhibition and apoptosis of human prostate cancer cells. For human hepatic microsomes in vitro, this led to inhibition of cytochrome P450 3A4-mediated metabolism and inactivation of 1,25D3. Of the several cytochrome P450 hydroxylating enzymes that are involved in metabolizing 1,25D and analogs, cytochrome P450 24A1 (CYP24A1) is primarily considered. The expression of CYP24A1 mRNA is used as a measure of the transactivating activity of a vitamin D analog. We have already determined the metabolic conversion of 1,25D analogs to CYP24A1 using the membrane fraction from recombinant *Escherichia coli* cells that expressed *h*CYP24A1 [25,26]. The crystal structure of rat recombinant CYP24A1 (Δ2-32, S57D mutant) has been reported (SSRL BL9-2 and BL12-2) [27,28], but the structure of the native full-length human CYP24A1 is still lacking. Therefore, taking-into-account the uncertain results from the modeling of analog binding to the pocket of the recombinant VDR, we opted for the modeling of metabolic resistance 1,25D analogs to the native cytochrome P450 3A4 (CYP3A4). The crystallographic 3D structure of human CYP3A4, as solved [29], conforms with the protein fold that is typical for the cytochrome P450 superfamily. Our approach is additionally supported by the very recent finding that the docking of peptidomimetic ligands to the cysteine-like protease of SARS-CoV-1 3CLpro (of the known 3D structure) facilitated the design of potent inhibitors with antiviral potency against SARS-CoV-2 3CLpro (of unknown 3D structure) [30]. Therefore, we postulated that by using the 3D structure of the native human CYP3A4 we would be able to predict the resistance of our new vitamin D analogs to CYP24A1 metabolism.

To this end, we used, for the first time, the 3D crystallographic data of cytochrome CYP3A4 (RCSD PDB: 2V0M.pdb) [31,32] for our theoretical simulations of vitamin D analogs. CYP3A4 is a hepatic microsomal 24-hydroxylase of 1α-hydroxyvitamin D_2_ and its analogs [33], and is also a vitamin-25 hydroxylase. We have previously shown that the inducible enzyme CYP3A4 is also a source of oxidative metabolism of 1,25D3 in the human liver and small intestine [33,34].

CYP3A4 is multifunctional and also metabolizes endobiotics and xenobiotics [35]. This enzyme is responsible for the deactivating hydroxylation of ca. 50% of drug substances [29,36]. Therefore, we used CYP3A4 in our study because we were interested in the metabolic stability of our vitamin D-based potential drug candidates. Additionally, both CYP24A1 and CYP3A4 show the same monooxygenase activity and catalyze the side-chain oxidation of the hormonal form of vitamin D to the same side-chain hydroxylated metabolites [37]. In this study, we provide unprecedented evidence that the metabolic conversion of analogs of 1,25D2 by CYP24A1 can be correlated with the free enthalpy of binding of the analogs to CYP3A4. By using the 3D structural data of CYP3A4 as a starting parameter, we have been able to explain the differences in the metabolic resistance of the side-chain geometric analogs of 1,25D. We have also been able to predict the metabolic resistance of structurally related analogs of 1,25D2 with a branched side-chain, and in turn, direct the design and synthesis of vitamin D analogs toward more promising drug candidates.

The interactions of 1,25D analogs with CYP3A4 were elucidated by computational approaches which included molecular docking, molecular dynamics simulations, binding free enthalpy calculations, and density functional theory (DFT) calculations. The relatively large active site of CYP3A4 shows large flexibility, allowing for side-chain fluctuations and the presence of various functional groups. There is also a large malleability that allows the adoption of a different shape depending on the ligand structure; making CYP3A4 a challenging protein to simulate [38]. Based on our experience of simulating molecular mechanisms for similar systems [39,40], simulations of CYP3A4 complexed with vitamin D analogs were carefully compared in terms of their structural and energetic properties. Their molecular structures were used to explore the possible binding modes with the amino acids of the active site of CYP3A4, as well as to show their different capacities for interaction with heme groups, which has biological consequences. Our calculations were compared with the previously determined metabolic resistance of analogs of 1,25D2 against the CYP24A1 hydroxylating enzyme. In order to check the extent of the correlation observed for the series of structurally related 1,25D2 analogs, preliminary studies were performed using two analogs of 1,25D3 that have very different structures.

## 2. Results and Discussion

### 2.1. Potential Binding Site of 1,25D Analogs in CYP3A4

**Figure 2:**
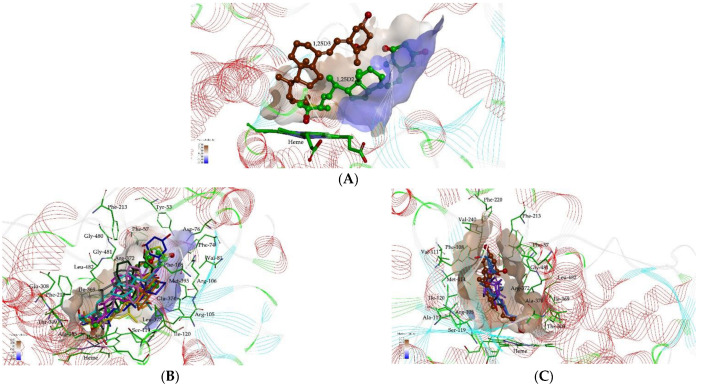
Close-up view of an active site of the CYP3A4 structure. (**A**) View of the binding of 1,25D2 (green) and 1,25D3 (brown) in the active site CYP3A4. (**B**,**C**) View of 1,25D2 and 1,25D3 analogs in the pocket of CYP3A4. Predicted superposition of compounds. (**B**) 1,25D2 (C atoms shown as green), PRI-5106 (C atoms shown as dark green), PRI-5105 (C atoms shown as magenta), PRI-1916 (C atoms shown as cyan), PRI-1917 (C atoms shown as pink), PRI-1906 (C atoms shown as blue), PRI-1907 (C atoms shown as grey), PRI-5201 (C atoms shown as yellow), and PRI-5202 (C atoms shown as orange). (**C**) PRI-1901 (C atoms shown as purple) and PRI-2205 (C atoms shown as blue). Surface hydrophobicity is depicted by the shaded colors: negative values (blue) correspond to hydrophilic residues, whereas positive values (brown) correspond to hydrophobic residues.

Molecular docking studies were carried out to predict the binding site and the orientation at the active site of CYP3A4 for ten analogs of 1,25D (eight analogs of 1,25D2 and two analogs of 1,25D3). The orientation and residue bonding of ketoconazole (as a reference inhibitor for the testing of the binding affinities to the CYP3A4) were inferred from the X-ray structure of human CYP3A4 (PDB entry 2V0M). This served as a model for the simulations of the interactions of vitamin D analogs with the enzyme. We supposed that the size of the molecular structure of vitamin D analogs was comparable to that of ketoconazole, and assumed that the docking of 1,25D analogs into the activity domain did not significantly perturb the crystal structure of the 2V0M. To validate the suitability of the selected docking model, we first re-docked ketoconazole to ensure that its bonding to the CYP3A4 binding site was consistent with the original structure of 2V0M. According to the ketoconazole-docking simulation, the complex showed that eight significant amino acids are involved in the interactions: Leu-210, Phe-241, Ile-301, Ala-305, Ala-370, Arg-372, Gly-481, and Leu-482, as well as the heme group, and are indispensable for the activity of the classic inhibitors. Next, all of our analogs were docked to the CYP3A4 pocket. The analogs effectively filled the active site cavity of CYP3A4. The position of their 25-hydroxyl was located close to the heme ring, indicating that it might be the potential metabolic site (see Figure 2).

As shown in Figure 2B,C, the inner wall of the pocket of CYP3A4 is formed by hydrophilic side chains (Asp-76, Arg-105, Arg-106, Ser-119, Thr-309, Arg-372, and Glu-374) and a hydrophobic region (formed by Phe-57, Phe-108, Ile-120, Leu-211, Ile-301, Phe-304, Ala-305, Ile-369, Ala-370, Leu-373, Gly-481, Leu-482, and Hem-499). The predicted location of 1,25D analogs in the CYP3A4 pocket is different for the 1,25D2 and 1,25D3 analogs (see Figure 2A). The 1,25D2 analogs interacted with the CYP3A4 pocket using hydrogen bonds and hydrophobic and van der Waals interactions, while the interaction of 1,25D3 and its analogs were mainly hydrophobic in nature. To examine the stability of the 1,25D analogs bound at the active site of CYP3A4, MD simulations were performed on the CYP3A4-liganded analog complex.

### 2.2. Importance of Side-Chain Geometry of 1,25D2 Analogs in the CYP3A4 Binding Site

**Figure 3:**
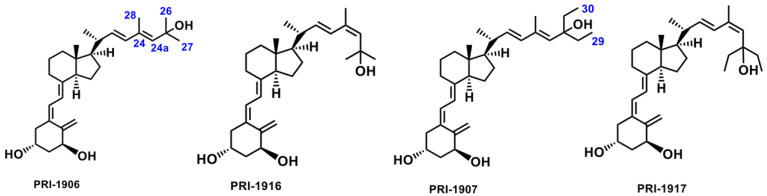
Structural formulas of the analogs of 1α,25-dihydroxyvitamin D_2_ (PRI-1906, PRI-1907, PRI-1916, and PRI-1917).

The structural formulas of PRI-1906 and PRI-1916, and PRI-1907 and PRI-1917 (Figure 3) are quite similar, but the extent to which they bind to CYP24A1 is different. Experimental data indicated that the binding to CYP24A1 of the C-25 dimethyl analog PRI-1916 of (24Z) geometry was much lower than that of the (24E)-25-dimethyl analog PRI-1906. Similarly, the binding of (24Z)-25-diethyl analog PRI-1917 was lower than that of the respective (24E)-25-diethyl analog PRI-1907 [33].

**Figure 4:**
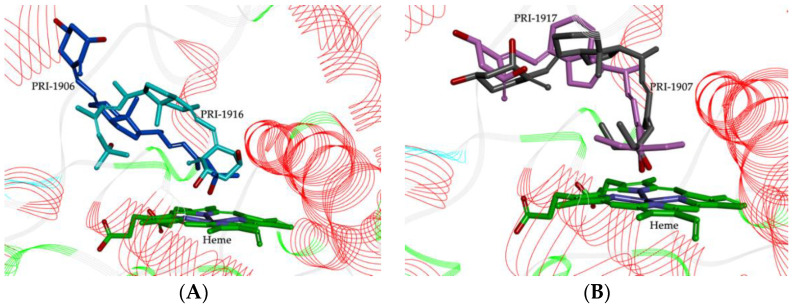
MD simulated conformational differences between 1,25D2 analogs located above the heme ring. (**A**) PRI-1906 (C atoms shown as blue) and PRI-1916 (C atoms shown as cyan); (**B**) PRI-1907 (C atoms shown as grey) and PRI-1917 (C atoms shown as pink).

Theoretical calculations showed that the geometry of the side-chain of the series of four analogs of 1,25D2 also followed the same trend as the predicted stereoselective activity of cis/trans isomers in the human CYP3A4 model. The MD resulting orientations of analogs, presented in Figure 4, define the geometric preferences of the four compounds on the active site of CYP3A4.

**Figure 5:**
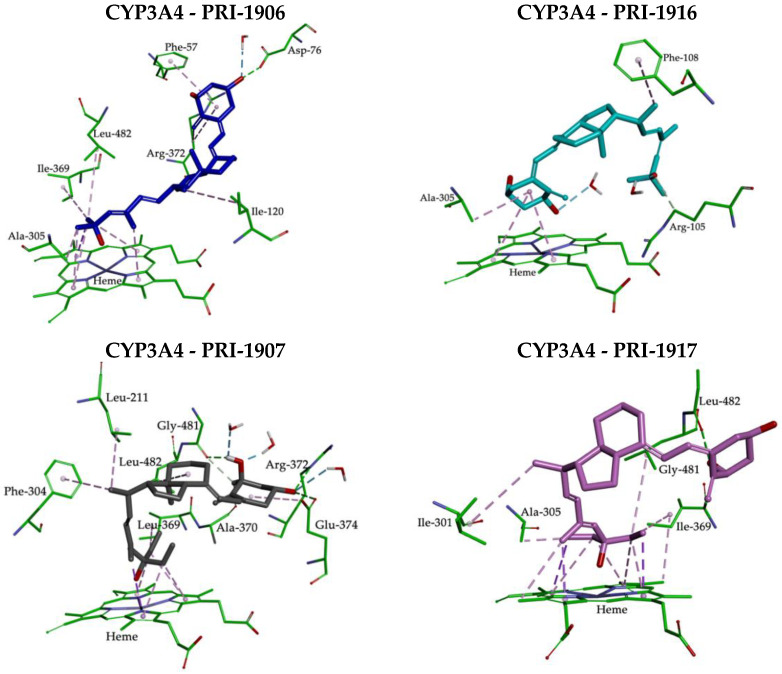
Molecular interactions between 1,25D2 analogs (PRI-1906, PRI-1907, PRI-1916, and PRI-1917) and CYP3A4 resulting from MD simulations. Hydrophobic interactions are shown as magenta dashed lines and hydrogen bonds as green.

Analogs PRI-1906 and PRI-1916 bind to the active site in different ways and with different affinities. Due to the side-chain geometry, PRI-1916 was predicted to adopt the U-shaped conformation in the CYP3A4 pocket, while (24E)-25-dimethyl PRI-1906 adopted an extended conformation. The most noticeable difference was that the U-shaped side-chain A-ring of PRI-1916 was rotated by 180° to the heme group, compared with the extended side-chain of the A-ring of PRI-1906 (Figure 4A). This could explain the lower binding of PRI-1916 for CYP3A4. PRI-1916 was not able to form a significant portion of the hydrophobic interactions as for PRI-1906 and with residues Phe-57, Ile-120, Ala-305, Ile-369, Leu-482, and Arg-372. On the other hand, the A-ring 3-hydroxyl of PRI-1906 engaged in a strong hydrogen bond at the binding site with the Asp-76 residue (see Figure 5 and Figure S1 in Supplementary Materials). For PRI-1907/CYP3A4 and PRI-1917/CYP3A4 complexes, the position of their 25-hydroxyl was almost the same in the active site of the receptor (Figure 4B), but the presence of the (24E)-25-diethyls of PRI-1907 affected the parallel arrangement of A- and CD-rings to the heme group.

The parallel arrangement of the A- and CD-rings to the heme group is conducive to the formation of very strong hydrogen bonds by the A-ring hydroxyls with the amino acid residues Ala-370, Arg-372, Glu-374, and Gly-481 in CYP3A4 (see Figure 5 and Figure S1 in Supplementary Materials). Furthermore, the 1- and 3-hydroxyl of PRI-1907 formed hydrogen bonds with water molecules. In contrast, the docked analog PRI-1917, with (24Z)-25-diethyls in the side-chain, showed the perpendicular arrangement of A- and CD-rings to the heme group that caused only the 1-hydroxyl to be involved in a hydrogen bond with amino acid residue Gly-481. This could lead to a lower affinity of PRI-1917 for the CYP3A4 cavity compared to that of PRI-1907 (see Figure 5 and Figure S1 in Supplementary Materials).

### 2.3. Molecular Interactions between the CYP3A4 Binding Site and 1,25D2 Analogs

**Figure 6:**
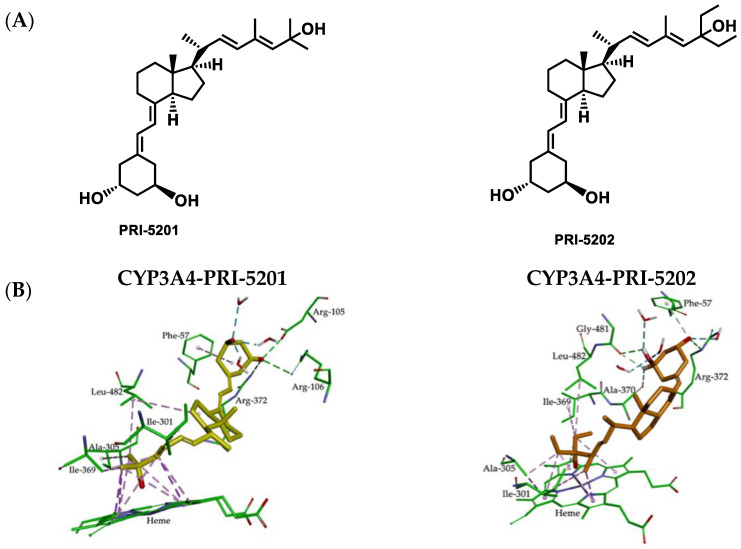
(**A**) Structural formulas of the analogs of 1α,25-dihydroxyvitamin D_2_ (PRI-5201 and PRI-5202). (**B**) Molecular interactions between 1,25D2 analogs (PRI-5201 and PRI-5202) and CYP3A4 resulting from MD simulations. Hydrophobic interactions are shown as magenta dashed lines and hydrogen bonds as green.

**Figure 7:**
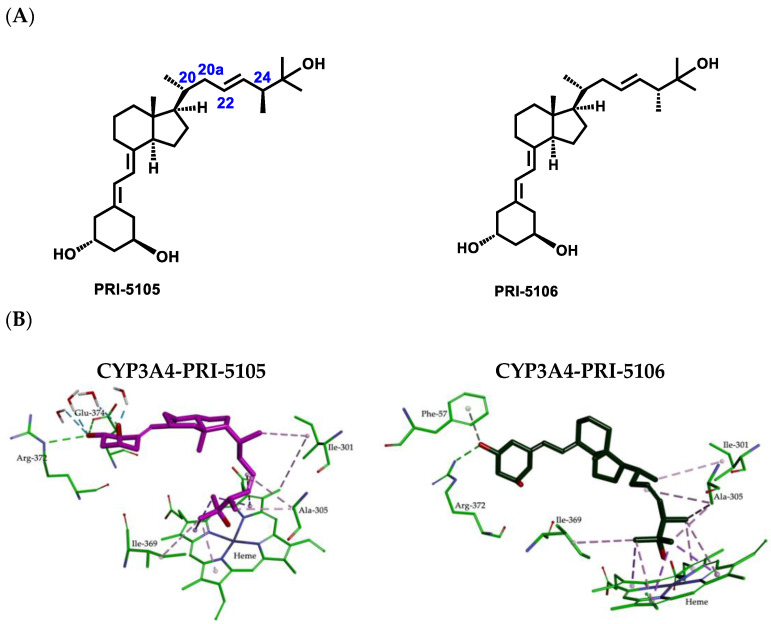
(**A**) Structural formulas the analogs of 1α,25-dihydroxyvitamin D_2_ (PRI-5105 and PRI-5106). (**B**) Molecular interactions between 1,25D2 analogs (PRI-5105 and PRI-5106) and CYP3A4 resulting from MD simulations. Hydrophobic interactions are shown as magenta dashed lines and hydrogen bonds as green.

MD simulations of the CYP3A4-vitamin D analog complexes were performed to further investigate the binding of ligands and the effect of their structures on the intermolecular interactions. The MD resulting orientations of analogs, presented in Figure 5, Figure 6, Figure 7 and Figure S2 in Supplementary Materials, define the key interactions in the active site of the human CYP3A4 model. The binding free enthalpy values, calculated using the MM-PBSA method, are shown in Table 1, together with the experimental metabolic conversions by CYP24A1. A correlation was observed between the experimental metabolic conversion by CYP24A1 and the theoretically estimated binding free enthalpy of the CYP3A4-ligand complexes (ΔG_bind_) (see Figure S3 in Supplementary Materials). The correlation coefficient, equal to R^2^ = 0.853, confirmed that the calculated enthalpy of binding to the vitamin D hydroxylating enzyme CYP3A4, with the crystal structure solved, shows good agreement with the experimental metabolic conversion by the main vitamin D hydroxylating enzyme CYP24A1. In other words, the experimental metabolic conversion of an analog by CYP24A1 advantageously decreases with the decrease in the calculated free enthalpy of the binding of an analog of 1,25D2 to CYP3A4.

The lowest value for the ΔG_bind_ enthalpy was obtained for PRI-1907. This is most likely a consequence of a good fit, as mentioned above, and the formation of very strong hydrogen bonds with amino acids that have an important role in the CYP3A4 active site. The analog of PRI-5202, which lacks a 19-methylene, showed a similarly folded conformation in the pocket of CYP3A4 but its location was rather different (see Figure S2A in Supplementary Materials). Apart from the typical interactions, the side-chain of PRI-5202 allowed for favorable interactions of the 28-methyl with Ile-301 and 26, 27-methylene with amino acid residues Ala-305, Ile-369, and Leu-482. The A-ring 3-hydroxyl was involved in additional hydrogen bonds with the Phe-57 residue (Figure 6 and Figure S1 in Supplementary Materials). Importantly, PRI-5202 is also highly solvated by water molecules that are present inside the pocket of CYP3A4.

The relatively high values of enthalpy obtained for both PRI-5105 and PRI-5106 result from the presence of an additional 28-methyl at C-24, irrespective of the (24*S*)-or (24*R*)-absolute configuration. PRI-5105 and its 24-diastereomer PRI-5106 were predicted to be accommodated in the CYP3A4 pocket in twisted conformations (see Figure S2B in Supplementary Materials). These conformations are energetically unfavorable and decrease the hydrophobic interactions with the surrounding amino acids (see Figure 7). The molecular flexibility conferred by one carbon extension (C-20a) to the side-chain of PRI-5105 and PRI-5106 results in a decreased metabolic resistance, emphasizing the importance of side-chain rigidity and the correct position in the active site. Notably, these analogs showed relatively low resistance to CYP24A1-dependent metabolic conversion (Table 1). The MD simulation showed that the location within the cavity of analogs PRI-5105 and PRI-5106 is largely the same, and the molecules are surrounded purely by the interior pocket residues of Phe-57, Ile-301, Ala-305, Ile-369, Arg-372, and Glu-374 which create hydrophobic interactions and hydrogen bonds.

Compared to PRI-1906, PRI-5201 has the 19-*nor* modification and the PRI-5201/CYP3A4 complex showed an extended conformation, as seen for the PRI-1906/CYP3A4 complex, but the position of 1-and 3-hydroxyls at the A-ring of PRI-5201 formed a very strong hydrogen bond with Asp-76, Arg-106, Arg-372, and three water molecules (see Figure 6 and Figure S1 in Supplementary Materials). This is likely responsible for the higher affinity of PRI-5201, versus that of PRI-1906.

### 2.4. Metabolic Conversion of 1,25D3 Analogs PRI-1901 and PRI-2205 to hCYP24A1-Mediated Degradation

**Figure 8:**
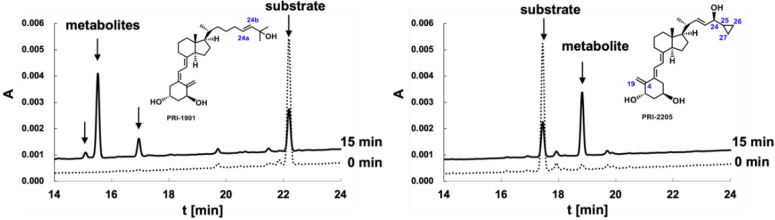
HPLC profiles of 1,25D3 analogs (PRI-1901 and PRI-2205) and their metabolites, before and after incubation with *h*CYP24A1. UV absorbance was recorded at 254 nm. The upper chromatograms represent profiles of the reaction mixture following incubation with *h*CYP24A1 for 15 min. The lower chromatograms were obtained at the starting point.

The membrane fraction prepared from the recombinant *Escherichia coli* cells that expressed *h*CYP24A1 [25,26] was used to examine the metabolism of 1,25Ds, as previously done for the 1,25D2 analogs. The HPLC metabolic profiles of PRI-1901 and PRI-2205 are shown in Figure 8.

The metabolic conversion of PRI-2205 was 44 ± 6% and the same as that observed for the parent 1,25D3 [10]. This suggests that modifications such as introducing an isolated double bond in the regular vitamin D_3_ side-chain at C-22 as well as the formal transfer of the 19-methylene from C-10 to C-4 position in the A-ring do not improve the metabolic resistance of analogs. Quite unexpectedly, the extension of the side-chain by two carbon units in PRI-1901 increased the metabolic conversion up to 58 ± 9%.

### 2.5. Molecular Interactions between CYP3A4 Binding Site and 1,25D3 Analogs

**Figure 9:**
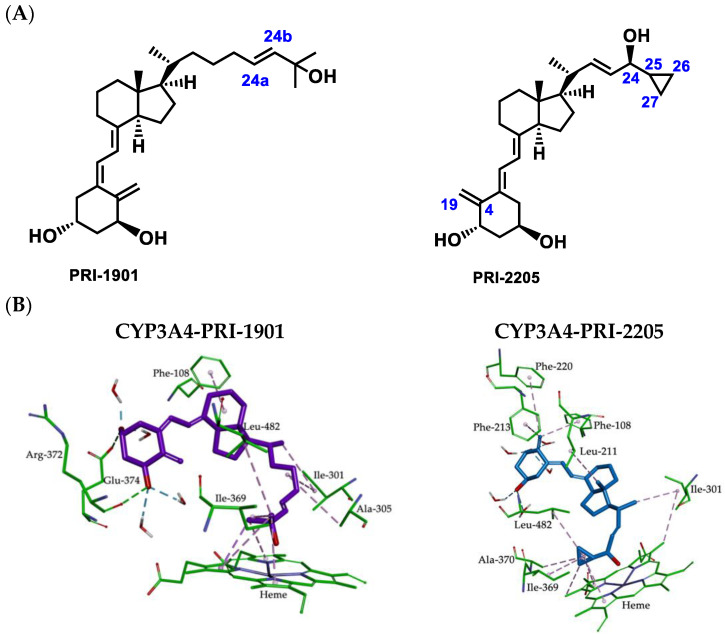
(**A**) Structural formulas of the analogs of 1α,25-dihydroxyvitamin D_3_ (PRI-1901 and PRI-2205). (**B**) Molecular interactions between 1,25D3 analogs (PRI-1901 and PRI-2205) and CYP3A4 resulting from MD simulations. Hydrophobic interactions are shown as magenta dashed lines and hydrogen bonds as green.

Based on the experimental data obtained for the metabolic conversion of 1,25D3 analogs by the CYP24A1 enzyme, we examined the nature of the mode of action that can be created between analogs and the CYP3A4 enzyme. This was investigated for the PRI-1901 and PRI-2205 analogs of 1,25D3 by using the human CYP3A4 model (see Figure 9). The calculated binding free enthalpy values for PRI-1901 and PRI-2205 were −62 kcal/mol and −64 kcal/mol, respectively (compared to −29 kcal/mol for 1,25D3). The interactions inside the complexes of PRI-1901 and PRI-2205 are shown in Figure 9 and Figure S4 in the Supplementary Materials.

Analog PRI-1901, which has a (24a*E*) double bond that is located in the vicinity of the C-25 center in the side-chain, is more flexible and adopts a U-shaped conformation. This does not allow for the correct disposition in the active site. The binding model for the PRI-1901 and CYP3A4 protein revealed that the 1,25D3 analog forms two hydrogen bonds with the two residues of the active site of CYP3A4 (Arg-372 and Glu-374) and that there are hydrophobic interactions with the amino acid residues Phe-108, Ile-301, Ala-305, Ile-369, and Leu-482. On the other hand, the higher affinity of PRI-2205 can be attributed to its extended conformation and may also be due to the presence of the cyclopropyl ring on the C-25 carbon atom at the side-chain which forms hydrophobic interactions with the amino acid residues Ile-369, Ala-370, and Leu-482 as well as with the heme ring. It can be seen (Figure 9) that the 19-methylene at the C-4 position makes strong π-alkyl interactions with the key residues of the active site of CYP3A4, such as the aromatic ring of Phe-108, Phe-213, and Phe-220. Furthermore, the positions of the A-ring hydroxyls of PRI-2205 allow the formation of a network of hydrogen bonds with water molecules which stabilize the binding of the PRI-2205 in the active site of CYP3A4.

## 3. Materials and Methods

### 3.1. Theoretical Calculations

#### 3.1.1. Dataset Preparation

In this study, we made use of our previously published and new experimental data for the metabolic resistance of analogs of 1,25D2 (PRI-5106, PRI-5105, PRI-1916, PRI-1917, PRI-1906, PRI-1907, PRI-5201, and PRI-5202) and 1,25D3 (PRI-1901 and PRI-2205) [16,41,42]. The starting conformations of the analogs were constructed based on the solid-state diffraction data of structurally related compounds to eliminate any subjectivity in generating the three-dimensional structure [43]. The molecular structures of the 1,25D analogs were optimized using the density functional theory (DFT) at the B3LYP/6-311^+^G(d,p) level implemented in the Gaussian 16 program [44]. The electrostatic potential (ESP) of the atomic partial charges of the atoms was computed using the Breneman model [45] which reproduces the molecular electrostatic potential. The crystal structure of human CYP3A4 (PDB entry 2V0M) was obtained from the RCSB Protein Data Bank [32]. The PDB file presents a co-crystal with a ketoconazole ligand in the active site. Ligand (except for the heme group), water molecules, and inorganic ions were removed before making the calculations, and hydrogen atoms were added to reflect the physiological pH. Prior to the analysis, the iron atom in the heme group was constrained to preserve its bonding to the nitrogen atoms of the heme after the application of the CHARMm force field.

#### 3.1.2. Molecular Docking and Dynamic Simulation

Docking and molecular dynamic (MD) simulations were performed using the Discovery Studio 2020 with a visual interface BIOVIA [46]. To identify the starting structures for the subsequent computations of the binding affinity of 1,25D analogs for CYP3A4; a rigid docking procedure was performed using the CDOCKER protocol of Discovery Studio 2020. The active site was defined with a radius of 15 Å around the analog present in the 2V0M crystal. The analogs were allowed to interact with the residues within the binding site spheres to generate ten conformations. The best poses predicted by CDOCKER were used as the starting points in the MD simulation.

MD simulations were run using the CHARMm force field [47] implemented in the module of Discovery Studio 2020. The molecular parameters and atomic charges for the protein were taken from a set of CHARMm force field parameters. Each model of the CYP3A4-analog complex was inserted into a cubic box of water molecules (TIP3P models) [48] extending up to 10 Å from any solute atom. Counter-ions (Na^+^, Cl^−^) were added randomly to each complex at a concentration of ~0.15 M, close to physiological conditions, by using the Solvation Module of Discovery Studio 2020. All energy minimization and MD simulations were performed using the Particle Mesh Ewald (PME) algorithm [49] for the correct treatment of electrostatic interactions [50] and periodic boundary conditions. Prior to MD simulations, all systems were minimized based on the steepest descent method with 3000 steps followed by 3000 conjugate gradient energy-minimization steps (until the RMS gradient of the structure was below 0.01 kcal/mol·Å) with an applied restraint potential of 10 kcal/mol·Å^2^ for protein. The conjugate gradient algorithm without restraint was further carried out with an additional full minimization of 1000 steps. A gradual heating MD simulation from 50 to 300 K was executed for 50 ps. Following heating, an equilibration estimating 100 ps of each system was conducted (the operating temperature was kept constant at 300 K). In the stages of heating and equilibration, the protein was fixed with a force constant of 5 kcal mol^−1^ Å^−2^. Using two phases, the equilibrated system was taken as the starting structure for production runs. In the first phase, NVT was performed at 300 K for 10 ns in the presence of a weak harmonic restraint on the solute, and in the second phase, NPT at 300 K and 1 bar for 10 ns. During the 20 ns simulations, snapshots were stored every 1 ps and used for the analysis. All simulations were run with SHAKE on hydrogen atoms with a 2 fs time step and Langevin thermostat for temperature control [51]. The integration time step was set to 1 fs. The cut-off distance for non-bonded terms was set to 10 Å.

#### 3.1.3. Binding Free Enthalpy Calculation

Even though docking combined with MD simulations provides a clear image of the shape complementarity between the analog and the protein, there is the need for additional and essential information regarding the free enthalpy of binding. This assesses the affinity of an analog to its target. To compare the stability of each binding mode while considering solvent effects, we calculated the binding free enthalpy (ΔG_bind_) for each mode by using the MM/PBSA method in Discovery Studio 2020. The ΔG_bind_ of 1,25D analogs to CYP3A4 was calculated using Equation (1):

ΔG_bind_ = G_CYP3A4–analog_ − G_CYP3A4_ − G_analog_ (1)

where G_CYP3A4–analog_ is the free enthalpy of complex, G_CYP3A4_ is the free enthalpy of CYP3A4, and G_analog_ is the free enthalpy of tested analogs. Binding free enthalpy was calculated based on the average structures obtained from the 20 ns of MD trajectories.

### 3.2. Metabolic Conversion of 1,25D3 Analogs PRI-1901 and PRI-2205 to CYP24A1-Mediated Degradation

CYP24A1-mediated degradation of the two analogs of 1,25D3 (PRI-1901 and PRI-2205) was determined using recombinant human CYP24A1 (*h*CYP24A1) as described previously [25,26]. The reaction mixture containing 2.0 μM bovine adrenodoxin, 0.2 μM bovine adrenodoxin reductase, 20 nM CYP24A1, 5 µM 1,25D3 analog, 1 mM NADPH, 100 mM Tris-HCl (pH 7.4), and 1 mM EDTA in total volume of 100 mL, was incubated at 37 °C for 15 min. The reaction was quenched by the addition of four volumes of chloroform/methanol (3:1) and vigorous shaking. The organic phase was collected and dried down under reduced pressure. The residue was dissolved in acetonitrile and centrifuged at 20,000× *g* for 15 min. The supernatant (applied volume 40 μL) was submitted to HPLC Capcell-Pak C18 UG120 4.6 mm × 250 mm column (Phenomenex, Tokyo, Japan) at a flow rate of 1 mL/ according to previously described conditions [25,26]. Samples were eluted by linear gradients of water-acetonitrile 20–100% solution (0–25 min) followed by 100% acetonitrile (25–40 min). The eluted metabolites of 1,25D3 analogs were detected by UV absorbance at 254 nm. The amount of a 1,25D3 analog in the eluates was calculated from the peak area. The percent of metabolic conversion of the analog was calculated as a ratio of the peak area of metabolites to the sum of the peak areas of the remaining 1,25D3 analog and the metabolite or metabolites (assumed as 100%).

## 4. Conclusions

This work has shown for the first time how the mode of action of 1,25D analogs at the active site of CYP3A4 can be predicted using simulation models. The prediction is of high importance for vitamin D-based drug candidates as CYP3A4 deactivates ca. 50% of drug substances. The results of the computational analysis have revealed that the molecular interactions with the active site of the CYP3A4 enzyme were very different for the 25-dimethyl analogs PRI-1906 and PRI-1916, as well as for the 25-diethyl analogs PRI-1907 and PRI-1917. For PRI-1906 and PRI-1916, there was a head (A-ring) and tail (side-chain) inversion; while for PRI-1907 and PRI-1917 there was a completely different arrangement of the CD-ring system. Differences of the same kind might occur regarding the binding of the above analogs to the CYP24A1 enzyme. The differences in molecular interactions explain why pairs of analogs differing only in the (24*E*)-and (24*Z*)-geometry of the side-chain showed substantial differences in their metabolic resistance. The enzymatic conversion of (24*E*)-analogs PRI-1906 and PRI-1907 by CYP24A1 was relatively very low, while the conversion of (24*Z*)-analogs PRI-1916 and PRI-1917 was six times and ten-times higher, respectively. As we anticipated, the calculated free enthalpy of binding of a vitamin D metabolite or analog to CYP3A4 showed agreement with the experimental conversion of an analog by CYP24A1. The very low values obtained for the enthalpy for the analogs PRI-1906, PRI-1907, PRI-5201, and PRI-5202 correspond to the lowest values of metabolic conversion. Similarly, high values for the enthalpy of the analogs PRI-1916 and PRI-1917 correspond with their high metabolic conversion. Finally, the relatively high values of enthalpy for both 1,25D2 and 1,25D3 correspond well with their very high metabolic conversion. Quite unexpectedly, the predicted location of 1,25D analogs in the CYP3A4 pocket is different for 1,25D2 and 1,25D3 analogs. The 1,25D2 analogs interact with the CYP3A4 pocket using hydrogen bonds, and hydrophobic and van der Waals interactions, while the interaction of 1,25D3 and its analogs are mainly hydrophobic in nature. Our metabolic studies of the two series of analogs of 1,25D2 and 1,25D3 have revealed that the position at C-10 or C-4 and the absence of 19-methylene in the A-ring, as well as the modifications that retained the regular size of the side-chain, did not influence the metabolic resistance of the analog. Regarding the structural modifications of vitamin D, a combination of both an extension by one carbon unit and introducing a conjugated system of double bonds in the side-chain results in the most pronounced increase in the resistance of an analog to *h*CYP24A1-mediated degradation.

In summary, we have shown that the metabolic resistance of a vitamin D analog to the CYP24A1 degrading enzyme, of unknown 3D structure, can be estimated by the binding strengths of the analogs to the CYP3A4 enzyme, which is of known 3D structure. The metabolic conversion of vitamin D analogs by CYP24A1 can be related to the free enthalpy of binding to CYP3A4 only for analogs of 1,25D2 that have very similar structures. Accordingly, examination of the correlation for analogs of 1,25D3 requires an extensive series of analogs that have a similar structure.

## Figures and Tables

**Table 1:** The experimental metabolic conversion of 1,25D2 and its analogs by CYP24A1 [10,16,41] and the calculated free enthalpy of binding to CYP3A4.

Vitamin D Metabolite and Its Analogs	Metabolic Conversion by CYP24A1 (%) ± SD	Free Enthalpy of Binding to CYP3A4 * ΔG_bind_ (kcal/mol)
1,25D2	34 ± 5	−24
PRI-5106	26 ± 3	−37
PRI-5105	25 ± 2	−27
PRI-1916	13 ± 4	−52
PRI-1917	10 ± 1	−65
PRI-1906	1.9 ± 0.7	−76
PRI-1907	0.9 ± 0.1	−120
PRI-5201	1.9 ± 0.1	−91
PRI-5202	2.0 ± 0.2	−100

* Rounded to the absolute value.

## Data Availability

All relevant data are provided in the article.

## Supplementary Materials

The following supporting information can be downloaded at: https://www.mdpi.com/article/10.3390/ijms23147845/s1.
