# Supplementary material for: In Silico Prediction of the Metabolic Resistance of Vitamin D Analogs against CYP3A4 Metabolizing Enzyme
Source: Int J Mol Sci. 2022 Jul 16;23(14):7845. doi: 10.3390/ijms23147845 (PMC9322940; doi:10.3390/ijms23147845)
Supplement: Supplementary file 1 [file ijms-23-07845-s001.zip › ijms-1757305-supplementary.pdf]

International Journal of *Molecular Sciences*

MDPI

Supplementary Materials

In silico Prediction of the Metabolic Resistance of Vitamin D Analogs against CYP3A4 Metabolizing Enzyme

Teresa Żołek [1,*], Kaori Yasuda [2], Geoffrey Brown [3], Toshiyuki Sakaki [2] and Andrzej Kutner [4]

[1] Department of Organic Chemistry, Faculty of Pharmacy, Medical University of Warsaw, 1 Banacha, 02-097 Warsaw, Poland; tzolek@wum.edu.pl

[2] Department of Pharmaceutical Engineering, Toyama Prefectural University, Toyama 939-0398, Japan; kyasuda@pu-tayama.ac.jp (K.Y); tsakaki@pu-toyama.ac.jp (T.S)

[3] School of Biomedical Sciences, Institute of Clinical Sciences, College of Medical and Dental Sciences, University of Birmingham, Edgbaston, Birmingham B15 2TT, UK; g.brown@bham.ac.uk

[4] Department of Bioanalysis and Drug Analysis, Faculty of Pharmacy, Medical University of Warsaw, 1 Banacha, 02-097 Warsaw, Poland; andrzej.kutner@wum.edu.pl

* Correspondence: tzolek@wum.edu.pl; Tel.: +48-225720643

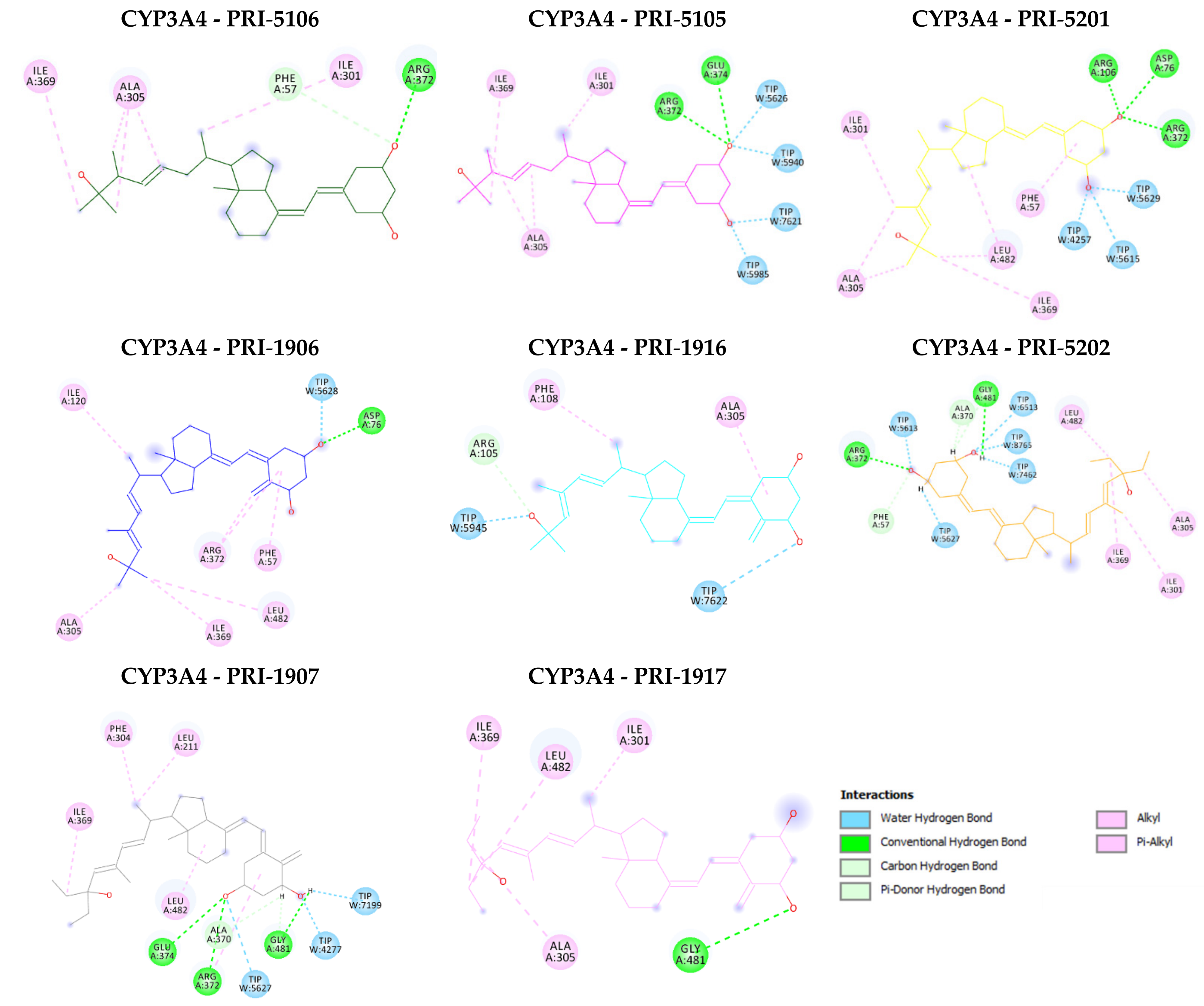

Figure S1. The 2D representation of the 1,25D2 analogs in the active site of CYP3A4.

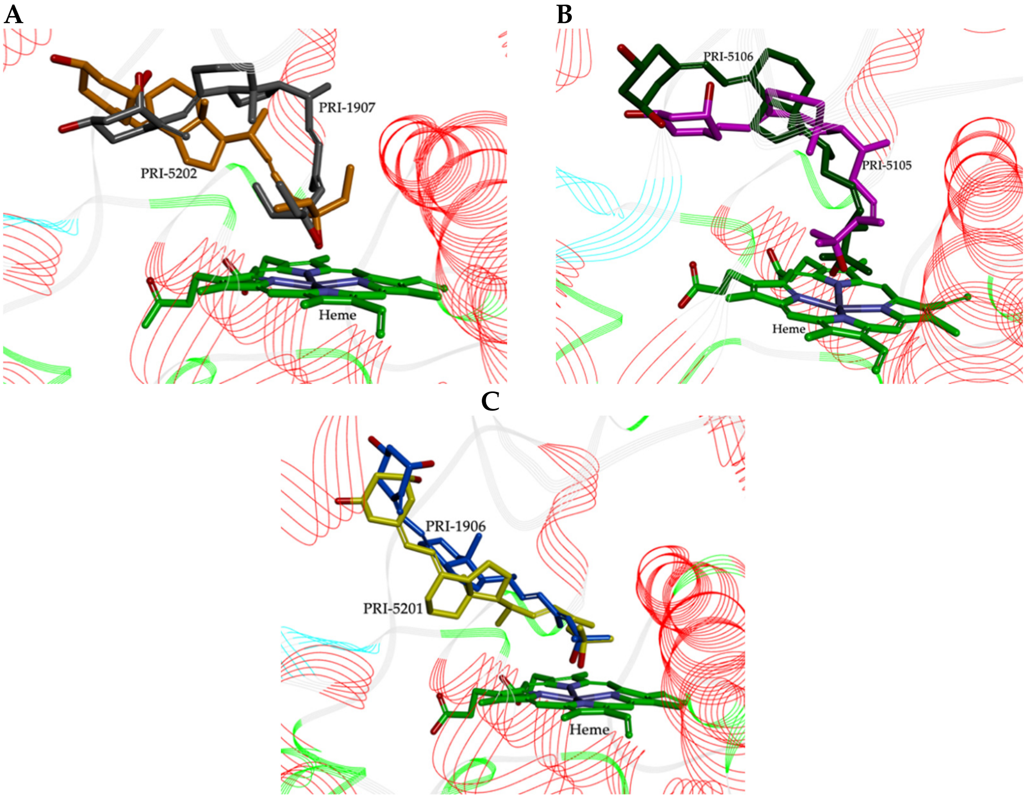

Figure S2. Illustration of conformational differences between 1,25D2 analogs which are located above the heme ring. (A) PRI-1907 (C atoms shown as grey) and PRI-5202 (C atoms shown as orange); (B) PRI-5105 (C atoms shown as magenta) and PRI-5106 (C atoms shown as dark green); (C) PRI-1906 (C atoms shown as blue) and PRI-5201 (C atoms shown as yellow).

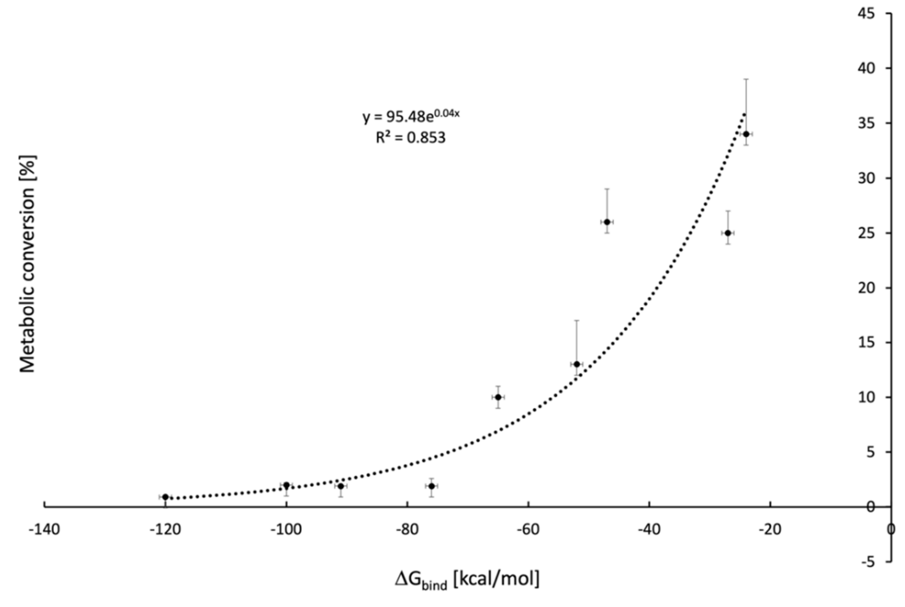

Figure S3. Correlation between the experimental metabolic conversion of 1,25D2 analogs by CYP24A1 and the free enthalpy of binding to CYP3A4. The exponential regression equation and its correlation coefficient are shown.

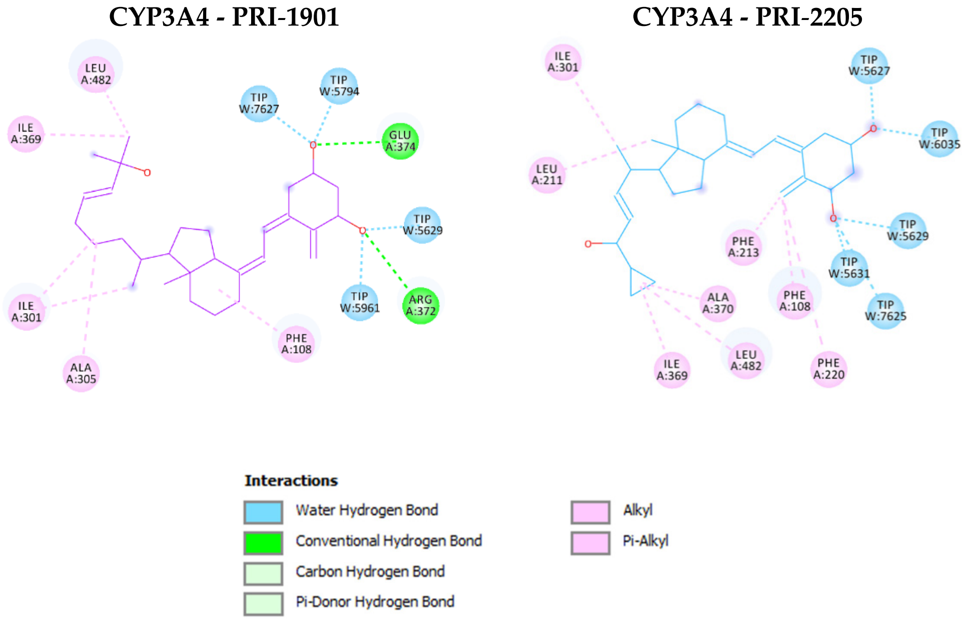

Figure S4. The 2D representation of the 1,25D3 analogs in the active site of CYP3A4.
